# A Compend of the Active Principles

**Published:** 1911-04

**Authors:** 


A Compend of the Active Principles—With Symptomatic Indications for their Therapeutic Use. By Harold Hamilton Redfield, A. B., M. D., Associate Professor of Therapeutics, Bennett Medical College, Chicago; Professor of Therapeutics and Physiology, Reliance Medical College, Chicago. The Clinic Publishing Co., Chicago. 1910. $1.00.

This little book is the concrete expression of an effort to epitomize in a condensed and usable form certain facts concerning some of the most valuable of the active-principle remedies. It does not attempt to tell all there is to tell about them, nor to cover the entire field of alkaloidal materia medica. This task has already been admirably attempted in larger works, notably in the Waugh-Abbott "Text-Book of Alkaloidal Therapeutics," but the author believes that there is, nevertheless, a place for a book of this size, which is based largely upon his own clinical experience. It was prepared with special reference to the needs of his students, whose interest in his lectures on active-principle therapeutics has served as an inspiration. He hopes that it may also prove of value to the rapidly growing class of progressive practitioners, who see in the alkaloidal movement the greatest promise for the future of our profession.

We cheerfully endorse and recommend Dr. Redfield's little book. —Daniel.

